# A Novel Analytical Model for Pore Volume Compressibility of Fractal Porous Media

**DOI:** 10.1038/s41598-019-51091-2

**Published:** 2019-10-09

**Authors:** Gang Lei, Nai Cao, Brian J. McPherson, Qinzhuo Liao, Weiqing Chen

**Affiliations:** 1 King Fahd University of Petroleum & Minerals, College of Petroleum Engineering & Geosciences, Dhahran, 31261 Saudi Arabia; 2 China University of Petroleum, College of Petroleum Engineering, Beijing, 102249 China; 3 University of Utah, Energy & Geoscience Institute, Salt Lake City, 84108 United States

**Keywords:** Energy harvesting, Applied mathematics

## Abstract

Over the past decades, many scholars have been studying the pore volume compressibility (PVC) of porous media. However, the fundamental controls on PVC of porous media are not yet definitive. Some scholars suggest a negative correlation between PVC and initial porosity, while others suggest a positive correlation. Motivated by this discrepancy, this paper presents a new analytical model to study the PVC of fractal porous media. The predictions are compared with test results and thereby validated to be accurate. In our attempt not only to complement but also to extend the capability beyond available models, the derived model accounts for multiple fundamental variables, such as the microstructural parameters and rock lithology of porous media. Results suggest that, there is a negative correlation between PVC and initial porosity, if all other parameters are fixed, the relationship between initial porosity and PVC is not monotonic. In addition, PVC decreases with rougher pore surfaces and smaller initial minimum pore radius. Besides providing theoretical foundations for quantifying PVC of porous media, this analytical model could be applied to estimate pore structure parameters of porous media using inverse modeling.

## Introduction

Pore volume compressibility (PVC) of porous media is a key parameter for characterizing coupled fluid transport and stress in reservoir rock^1–8^. PVC is of particular interest to the petroleum industry, especially for elastic energy evaluation, design, drilling and completion of wells, production forecasts, and material balance studies^2,9–13^. The earthquake seismology community also relies on PVC assessments for analysis of induced seismicity potential^14,15^.

For a porous media with pore volume *V*_*p*_, the PVC of porous media *c*_*p*_ may be determined as^13–20^:

1 $${c}_{p}=\frac{1}{{V}_{p}}\frac{\partial {V}_{p}}{\partial {p}_{p}}=-\,\frac{1}{{V}_{p}}\frac{\partial {V}_{p}}{\partial {p}_{eff}}$$

where *c*_*p*_ represents the pore volume compressibility (MPa^−1^), *V*_*p*_ represents the pore volume (cm^3^), *p*_*p*_ represents the pore pressure (MPa), and *p*_*eff*_ represents the effective stress (MPa).

In general, there are three traditional approaches to evaluate PVC including laboratory tests^16–26^, numerical methods^27,28^, and theory-based evaluation methods^20,29–32^. Probably the most common method is laboratory experimental testing, which may be classified into direct measurement methods and indirect measurement methods. In the specific content of the research described in this paper, relevant laboratory test methods, together with their characteristics, are summarized in Table 1.

**Table 1:** Laboratory experiment methods for pore volume compressibility.

Experiment models		Characteristics of the models
Direct measurement methods	Volume^20–23,33,34^	1. It’s a direct and simple measurement method. 2. The test is time-consuming, costly and may generate biased results without a systematic calibration.
	Mercury intrusion^19^	1. There is no micro interstice in the test process. 2. In combination with the N_2_ adsorption results and the mercury intrusion volumes, the compression of the matrix can be determined accurately.
Indirect measurement methods	Sonic velocity^24^	It can be used to predict PVC under reservoir conditions.
	Permeability test^25,26^	It can quantify the uncertainty.
Notes: Both direct measurement methods and indirect measurement methods are costly and time-consuming.		

The volume methods^20–23,33,34^ are probably the most commonly used measurement techniques to determine PVC. However, as stated in the literature^6,13,33,34^, during the experiments, the systematic errors resulting from deformation of pressurized parts (such as micro interstice between the sample and the sleeve in the core holder, core holder itself, connection pipes, etc.) exist and will affect the results. For example, according to some researchers^6^, the permeability difference between the first and second measurements in confining pressure loading-unloading processes varied much. Tarokh A.^34^ and Asaei H.^33^ also pointed out that the necessity of system calibration during the undrained and drained volumetric measurement tests. Specifically, the changes in loading/unloading process of confining pressure and pore pressure/effective stress during the tests lead to the change in the shape of the testing system (sleeve, core holder, pipe and other accessories) which make an extra change of volume especially for the jacketed drained tests. Physically speaking, this fluid volume change may cause a negative effect on the PVC of the porous media determination and increase the value of PVC. Although some researchers^6,33,34^ suggested using the systematic calibration tests to reduce this measurement error, the tests may make the volumetric measurement test process more time-consuming and costly. Thus, the test results of the volumetric method are usually costly and sometimes over-estimated especially without proper systematic calibrations. Nevertheless, it is worth noting that the repetitive stress treatment will also lead to changes in the pore structure of porous media. As an alternative direct measurement method, mercury intrusion method^19^ can be used to measure the PVC of porous media accurately, which eliminates the effects of micro interstice by avoiding using core holder. It is generally believed that, during the mercury intrusion porosimetry (MIP) measurements, one can easily observe the compression of samples as mercury intrusion pressure increases. The compression volume of porous media is the difference between the change of the mercury volume and the change of the pore filling volume (i.e., $\Delta {V}_{compression}=\Delta {V}_{mercury}-\Delta {V}_{pore-filling}$). Guo *et al*.^19^ conducted MIP tests on cylindrical coal samples with the diameter of 25 mm and the length of 50 mm, which were extracted from the middle of the coal blocks. And they crushed remaining blocks for N_2_ adsorption analyses. Results from MIP tests^19^ suggested there existed a linear relation between the mercury volume and intrusion pressure change when the pressure ranged from 7.35 MPa to 32.2 MPa (i.e., the ratio of $\Delta {V}_{mercury}$ and Δ*p* is a constant). Then, after determining the value of $\Delta {V}_{pore-filling}$ with N_2_ adsorption analysis, they estimated the value of $\Delta {V}_{mercury}/\Delta p$. Finally, the PVC of the coal matrix was determined by dividing $\Delta {V}_{mercury}/\Delta p$ by the matrix volume Δ*V*_*m*_. Besides the direct measurement methods^19–23^, many indirect measurement methods (e.g., sonic velocity method^24^ and permeability test method^25,26^) have been used for PVC determination. More details about the sonic velocity method and permeability test method can be found in the literature^24–26^.

By and large, the experiments are costly, and the test results are usually affected by experimental error. Due to the discrepancies in physical properties of core samples and experimental methods, there are many inconsistencies in the tests’ results. As an alternative, some effective numerical methods have been proposed to simulate fluid flow in reservoir rocks, and coupling PVC is important for such^27,28^. However, numerical methods demand computationally-intensive calculations and typically predicted results are subject to significant uncertainties, especially as the ones induced by numerical dispersion^29^, as well as other similar constraints^29,30^. Theoretical methods for assessment of PVC may be classified into empirical models^20,21,31,32^, analytic models^2,13^ and other complex models^35–37^. Hall^21^, Horn^31^ and Jalalh^32^ used empirical correlations to predict PVC. Based on matching experimental data of 12 samples (i.e., 5 sandstones and 7 limestones) with the porosity ranging from 2% to 26%, Hall^21^ derived the empirical correlation of PVC. As one of the earliest and primary formula, Hall’s correlation has been widely used in petroleum engineering. Newman^20^ compared the PVC data of 256 samples with porosity ranging from 1% to 35% (e.g., 197 sandstone samples and 59 limestone samples) with that from Hall’s correlation and found PVC predicted from Hall’s correlation was in poor agreement with the experimental data. Newman^20^ stated that PVC of various types of rocks with a certain porosity varied widely. In addition, Jalalh^32^ suggested that Hall’s correlation should be used with caution. Horn^31^ proposed the correlation of PVC for consolidated carbonates. However, the predicted results from Horn’s model were PVC of porous media with different lithologies under 75% of the lithostatic pressure. To avoid using the compilation published dataset (e.g., initial porosity and PVC) of rocks with various lithologies, Jalalh^32^ proposed a correlation for PVC prediction of carbonate rocks. Although the above correlations^21–23^ to some extent, can be applied to predict PVC of porous media, these correlations contain empirical parameters, which have no clear physical meaning and vary with reservoirs. Recently, Li *et al*.^2,13^ proposed an analytical model based on porosity and rock lithology parameters to calculate the PVC of the reservoir rock. Besides the empirical models and analytic models, some scholars also calculated the PVC using other complex models. Khatchikian^35^ analyzed the PVC of core samples using the Gassman equation, in which parameters were evaluated via geophysical log analysis. Poston and Chen^36^ and Yildiz^37^ analyzed rock compressibility with material balance and production data.

All of the above studies convince us that, limited by the complex pore structure, relevant theoretical methods for predicting PVC are very scarce. What’s more, some research results from different theoretical models are inconsistent with each other. For example, the PVC predicted by Li’s model is quite different from the values determined by the correlation proposed by Hall^21^. Specifically, Li’s model suggested that the larger the porosity is, the larger that PVC will be, which is a contrast to the conclusion from other studies^6,21,32^ that indicated a greater PVC corresponds to a reduced porosity. Additionally, Li *et al*.^2,13^ stated that the positive correlation between PVC and initial porosity was validated by the experiments conducted by de Oliveira *et al*.^18^ and Guo *et al*.^19^. However, after careful examination, we find that the results from de Oliveira *et al*.^18^ cannot validate Li’s model. The main reason is that the curve of PVC versus porosity was plotted at different pressure stages for each core, hence the results are not the same as those from Li *et al*.^2,13^. Furthermore, results from Guo *et al*.^19^ suggest that the PVC of porous media is affected by several factors, such as the initial porosity, moisture content, and macerals, etc. Though Guo *et al*.^19^ found the PVC of medium-volatile bituminous (mvb) and low-volatile bituminou (lvb) coals exhibited a positive correlation to initial porosity, they also found there was no correlation between the PVC and porosity for the high-volatile A bituminous (hvAb) coals. Moreover, Li’s model was based on the assumption that porosity is constant during deformation, which is questionable. In most cases, PVC is not just related to porosity, but also affected by other pore structure parameters^29,30,38^. To the best of our knowledge, previous researches did not relate the full complement of pore structure parameters to PVC, and the fundamental controls on PVC are not yet definitive.

As stated in the literatures, the interspaces in most of porous media have fractal characteristics^39–45^. Since Mandelbrot firstly introduced the concept of the fractal to describe self-similarity of fractal objects^39^, many studies have been conducted to study the fractal feature of porous media^40–45^. Many scholars have found and suggested that fractal geometry could be applied to well characterize the complex porous structure of porous media. As a result, we take the microstructure of porous media into account and derive an analytical solution to predict PVC, based on fractal theory. Compared with the previous studies, this new model doesn’t contain empirical constants. Furthermore, it can help to reduce the uncertainty in flow through porous media and obtain data with high accuracy. As the outline of this work, the analytical model for PVC is provided in the following section. Then, the results are analyzed, followed by the discussions of the derived model. Finally, the conclusions are presented.

## Methods

### Theoretical model for PVC

As suggested by decades of literature, the pore structure of reservoir rocks can be well described and quantified by fractal approach^39–45^. For the sake of simplicity, the cross-sectional shapes of pores in porous media are described by circles. Meanwhile, besides assuming capillary radius does not change in the direction of flow, Euclidean dimension should be assigned as 2 in this work. Then, with a fractal approach, the equations for pore surface area at zero stress *A*_*p*0_ (μm^2^) and pore volume before deformation *V*_*p*0_ (μm^3^) are^29,44,45^:

2 $$\{\begin{array}{rcl}{A}_{p0} & = & \frac{\pi {r}_{{\max }}^{{D}_{f0}}{D}_{f0}}{2-{D}_{f0}}({r}_{{\max }\,0}^{2-{D}_{f0}}-{r}_{{\min }\,0}^{2-{D}_{f0}})\\ {V}_{p0} & = & \frac{4\pi {r}_{{\max }}^{{D}_{f0}}{D}_{f0}}{3(3-{D}_{f0})}({r}_{{\max }\,0}^{3-{D}_{f0}}-{r}_{{\min }\,0}^{3-{D}_{f0}})\end{array}$$

where *r*_*max*0_ represents the initial maximum pore radius (μm), *D*_*f*0_ is the initial pore fractal dimension (dimensionless), *r*_*min*0_ represents the initial minimum pore radius (μm). The correlation of *D*_*f*0_ and the initial porosity ${\phi }_{0}$ (dimensionless) can be expressed as^27,43^:

3 $${\phi }_{0}={({r}_{{\min }0}/{r}_{{\max }0})}^{2-{D}_{f0}}$$

Affected by effective stress, the pore structure of porous media will change, and Eq. 2 may be rewritten as^29,43^:

4 $$\{\begin{array}{rcl}{A}_{p} & = & \frac{\pi {r}_{{\max }}^{{D}_{f}}{D}_{f}}{2-{D}_{f}}({r}_{{\max }}^{2-{D}_{f}}-{r}_{{\min }}^{2-{D}_{f}})\\ {V}_{p} & = & \frac{4\pi {r}_{{\max }}^{{D}_{f}}{D}_{f}}{3(3-{D}_{f})}({r}_{{\max }}^{3-{D}_{f}}-{r}_{{\min }}^{3-{D}_{f}})\end{array}$$

where *A*_*p*_ represents the pore surface area (μm^2^), *V*_*p*_ represents the pore volume (μm^3^), *r*_*max*_ represents the maximum pore radius (μm), *D*_*f*_ is the fractal dimension of pore after deformation (dimensionless), *r*_*min*_ represents the minimum pore radius (μm).

Assume that the specific surface area of porous media remains constant as effective stress increases, the following expression can be obtained as^29,46,47^:

5 $$\frac{{A}_{p0}}{{V}_{p0}}=\frac{3(3-{D}_{f0})}{4(2-{D}_{f0})}\frac{({r}_{{\max }0}^{2-{D}_{f0}}-{r}_{{\min }0}^{2-{D}_{f0}})}{({r}_{{\max }0}^{3-{D}_{f0}}-{r}_{{\min }0}^{3-{D}_{f0}})}=\frac{{A}_{p}}{{V}_{p}}=\frac{3(3-{D}_{f})}{4(2-{D}_{f})}\frac{({r}_{{\max }}^{2-{D}_{f}}-{r}_{{\min }}^{2-{D}_{f}})}{({r}_{{\max }}^{3-{D}_{f}}-{r}_{{\min }}^{3-{D}_{f}})}$$

By solving Eq. 5 for *D*_*f*_, the stress-dependent fractal dimension may be written as^29,47^:

6 $${D}_{f}=2-\frac{(2-{D}_{f0}){r}_{{\max }0}}{(3-{D}_{f0}){r}_{{\max }}-(2-{D}_{f0}){r}_{{\max }0}}$$

Combining Eqs 3 and 6, the stress-dependent porosity $\phi$ (dimensionless) is:

7 $$\phi ={({r}_{{\min }}/{r}_{{\max }})}^{\tfrac{(2-{D}_{f0}){r}_{{\max }0}}{(3-{D}_{f0}){r}_{{\max }}-(2-{D}_{f0}){r}_{{\max }0}}}$$

As discussed in a previous study^29^, the relationship between the stress-dependent equivalent pore radius *r* (μm) and effective stress *p*_*eff*_ is:

8 $$r={r}_{0}\{1-4{[\frac{3\pi (1-{v}^{2}){p}_{eff}}{4E}]}^{\beta }\}$$

where *r*_0_ is the equivalent radius before deformation (μm), *β* is the power law index (dimensionless), *E* represents the rock elastic modulus (GPa), and *ν* represents the Poisson’s ratio (dimensionless).

Based on Eq. 8, the following equation is:

9 $$\{\begin{array}{rcl}{r}_{{\max }} & = & {r}_{{\max }0}\{1-4{[\frac{3\pi (1-{v}^{2}){p}_{eff}}{4E}]}^{\beta }\}\\ {r}_{{\min }} & = & {r}_{{\min }0}\{1-4{[\frac{3\pi (1-{v}^{2}){p}_{eff}}{4E}]}^{\beta }\}\end{array}$$

Substituting Eq. 9 into Eq. 7, the stress-dependent porosity $\phi$ may be rewritten as:

10 $$\phi ={({r}_{{\min }0}/{r}_{{\max }0})}^{\tfrac{(2-{D}_{f0}){r}_{{\max }0}}{(3-{D}_{f0}){r}_{{\max }}-(2-{D}_{f0}){r}_{{\max }0}}}$$

Based on Eq. 1, the PVC *c*_*p*_ may be defined as^13^:

11 $${c}_{p}=-\,\frac{1}{{V}_{p}}\frac{\partial {V}_{p}}{\partial {p}_{eff}}=-\,\frac{1}{\phi }\frac{\partial \phi }{\partial {p}_{eff}}$$

Subsequently, the porosity correlation may be obtained through the integration of Eq. 11 ^20,32,38^ as follows:

12 $$\phi ={\phi }_{0}{e}^{-{c}_{p}{p}_{eff}}$$

Combining Eqs 10 and 12, the resulting expression relating porosity and effective stress is:

13 $$\mathrm{ln}\,(\frac{{\phi }_{0}}{\phi })=\frac{({r}_{{\max }0}-{r}_{{\max }})(2-{D}_{f0})(3-{D}_{f0})}{[(3-{D}_{f0}){r}_{{\max }}-(2-{D}_{f0}){r}_{{\max }0}]}\,\mathrm{ln}\,(\frac{{r}_{{\max }0}}{{r}_{{\min }0}})={c}_{p}{p}_{eff}$$

This relationship between $\mathrm{ln}({\phi }_{0}/\phi )$ and *p*_*eff*_ is linear, and the slope of the straight line represents PVC. By plotting the curve of $\mathrm{ln}({\phi }_{0}/\phi )$ and *p*_*eff*_, an estimate of PVC of porous media may be estimated with simple linear regression.

### Workflow of PVC determination

According to our proposed model, the suggested workflow for PVC determination may be summarized as follows:

Step 1: Based on the initial average porosity ${\phi }_{0}$ and initial average permeability *K*_0_ (μm^2^) of a porous medium, pore structural parameters, such as *r*_*max*0_, *r*_*min*0_, and the initial fractal dimension of pore *D*_*f*0_, may be determined using Eqs 3, 14 and 15 from the literature^29^,

14 $${K}_{0}=\frac{{2}^{{D}_{T0}}\pi {D}_{f0}{r}_{{\max }0}^{{D}_{f0}}}{16{A}_{0}{L}_{0}^{{D}_{T0}-1}}\frac{{r}_{{\max }0}^{3+{D}_{T0}-{D}_{f0}}-{r}_{c0}^{3+{D}_{T0}-{D}_{f0}}}{3+{D}_{T0}-{D}_{f0}}$$

15 $$\{\begin{array}{rcl}{A}_{0} & = & \tfrac{{A}_{p0}}{{\phi }_{0}}=\tfrac{\pi {r}_{{\max }0}^{2}{D}_{f0}}{2-{D}_{f0}}(\tfrac{{r}_{{\max }0}^{2-{D}_{f0}}}{{r}_{{\min }0}^{2-{D}_{f0}}}-1)\\ {L}_{0} & = & \sqrt{{A}_{0}}={r}_{{\max }0}\sqrt{\tfrac{\pi {D}_{f0}}{2-{D}_{f0}}(\tfrac{{r}_{{\max }0}^{2-{D}_{f0}}}{{r}_{{\min }0}^{2-{D}_{f0}}}-1)}\\ {D}_{T0} & = & 1+\tfrac{\mathrm{ln}\,\{\tfrac{1}{2}[1+\tfrac{1}{2}\sqrt{1-{({r}_{{\min }0}/{r}_{{\max }0})}^{2-{D}_{f0}}}+\tfrac{\sqrt{{[1-\sqrt{1-{({r}_{{\min }0}/{r}_{{\max }0})}^{2-{D}_{f0}}}]}^{2}+\tfrac{1}{4}[1-{({r}_{{\min }0}/{r}_{{\max }0})}^{2-{D}_{f0}}]}}{1-\sqrt{1-{({r}_{{\min }0}/{r}_{{\max }0})}^{2-{D}_{f}}}}]\}}{\mathrm{ln}\,[\tfrac{{D}_{f0}-1}{\sqrt{{D}_{f0}}}\sqrt{\tfrac{1-{\phi }_{0}}{4{\phi }_{0}}\tfrac{\pi }{2-{D}_{f0}}}\tfrac{{r}_{{\max }0}}{{r}_{{\min }0}}]}\end{array}$$

where *A*_0_ is the initial cross-sectional area of porous media (μm^2^). *L*_0_ is the initial capillary representative length (μm). *D*_*T*0_ is the initial tortuosity fractal dimension (dimensionless).

Step 2: Select parameters *E*, *v*, and the values of effective stress *p*_*eff*_, and then calculate the parameters *r*_*max*0_ and *r*_*min*0_ using Eq. 9. Then, the stress-dependent porosity $\phi$ may be determined with Eq. 10;

Step 3: Based on Eq. 13 and its plot, the PVC *c*_*p*_ may be estimated using linear regression.

## Results

### Model validation

As previously discussed, reports using analytical methods to study PVC are scarce^48^. Some typical correlations for PVC, together with their characteristics, are summarized in Table 2.

**Table 2:** Some correlations for PVC.

Author	Year	Model correlations for *c*_*p*_ (10^−4^ MPa^−1^)	Characteristics of the models
Hall^21^	1953	*c*_*p*_ = $2.587{\phi }_{0}^{-0.4358}$	1. The empirical relationships are proposed by non-linear regression curve fitting. 2. These models do not take rock lithology into account.
Horne^31^	1990	*c*_*p*_ = $1.4504{e}^{4.026-23.07{\phi }_{0}+44.28{\phi }_{0}^{2}}$	
Jalalh^32^	2006	*c*_*p*_ = $1.4504/(0.9574+0.3539{\phi }_{0}^{1.05})$	
Zhu^13^	2018	*c*_*p*_ = $0.3(1-2\nu ){\phi }_{0}/E$	1. It takes rock lithology into account. 2. It shows a positive correlation between PVC and ${\phi }_{0}$, which is against the results of other models.

**Figure 1:**
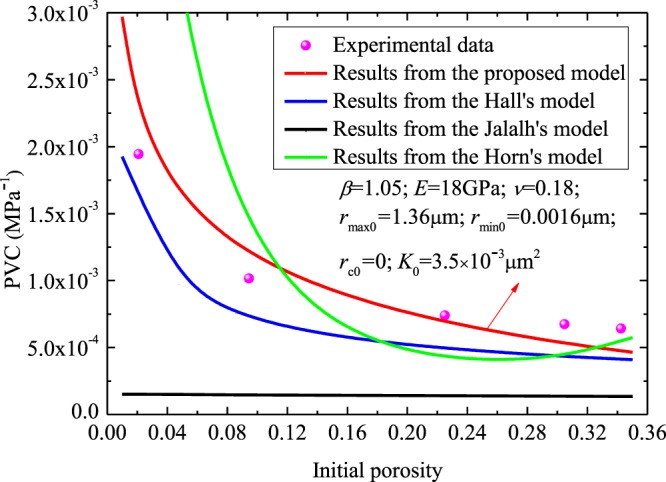
The test data^48^ versus PVC predicted from different models.

Figure 1 compares the measured PVC^48^ and that predicted by our derived model and other models in Table 2 (e.g. Hall’s model^21^, Horne’s model^31^ and Jalalh’s model^32^). In the experiment of da Silva Jr G.P. *et al*.^48^, the PVC tests were conducted on clean and dry rock samples with *K*_0_ of 3.5 × 10^−3^ μm^2^ and ${\phi }_{0}$ of 18.8%. In our proposed model, the parameter *E* assigned was 18 GPa, *ν* assigned was 0.18 and *β* assigned was 1.05. Besides, to ensure the parameter *K*_0_ is 3.5 × 10^−3^ μm^2^, the critical radius for irreducible water saturation assigned is 0, the initial porosity 18.8%, with the values of initial maximum and minimum radii at 1.36 μm and 0.0016 μm, respectively. Figure 1 reveals that the predicted PVC of our derived model is consistent with that determined by experimental data^48^, and our predictions are more consistent with experimental data than those predicted by previous models.

**Figure 2:**
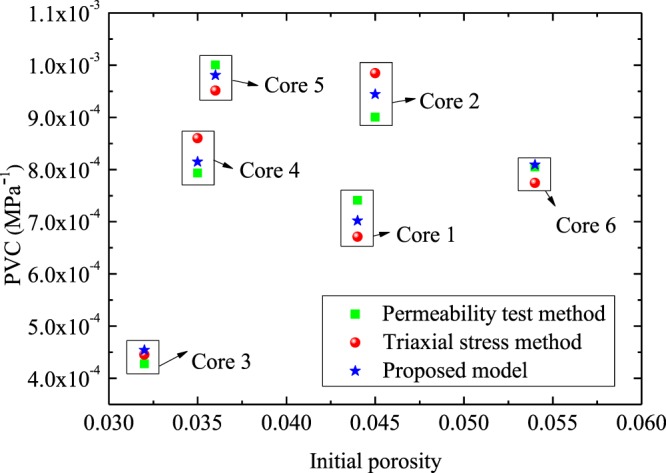
Experimental data^24^ versus the predictions: the results from different methods.

**Figure 3:**
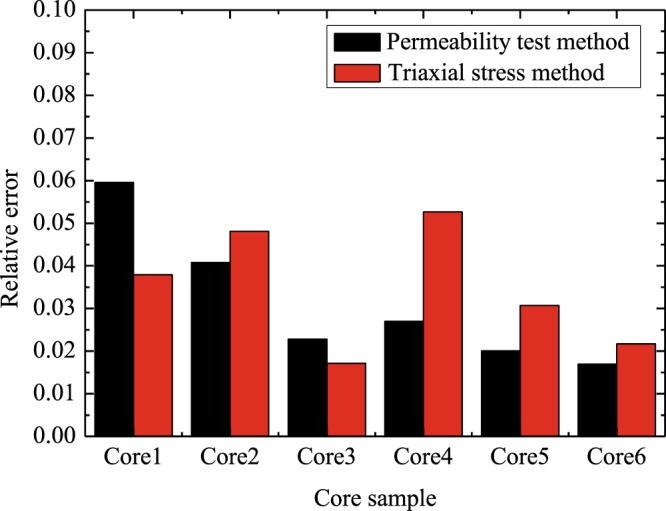
Experimental data^24^ versus the predictions: the relative error.

Figures 2 and 3 compares the PVC values predicted by the derived model with test data^24^. In the experiment of He *et al*.^24^, the PVC tests were carried out on six rock samples with a permeability test method and a triaxial stress method. The parameters applied in the calculations are summarized in Table 3. Since the relative error is less than 6.0%, results (Figs 2 and 3) show that the predicted results of our model are in agreement with the corresponding test data^24^. Results (see Fig. 2) also demonstrate that the PVC is not only related to ${\phi }_{0}$, but also is affected by other pore structure parameters. For example, the PVC of Core 1 with an initial porosity 4.4% is less than that of Core 4 with initial porosity 3.5%. However, the PVC of Core 4 with initial porosity 3.5% is larger than that of Core 3 with initial porosity 3.2%. Thus, the effects of pore structure parameters on PVC should probably be considered to elucidate primary controls on it.

**Table 3:** Parameters applied in the proposed model.

Core No.	*r*_*min*0_ (10^−5^ μm)	*r*_*max*0_ (10^−2^ μm)	*K*_0_ (10^−6^ μm^2^)	*φ*_0_ (%)	*E* (GPa)	*ν*	*β*
Core 1	2.0	2.35	0.26	4.4	28	0.18	1.109
Core 2	1.7	1.70	0.14	4.5	28	0.18	1.069
Core 3	5.0	2.32	0.18	3.2	28	0.18	1.195
Core 4	3.0	1.80	0.12	3.5	28	0.18	1.105
Core 5	5.0	2.50	0.24	3.6	28	0.18	1.080
Core 6	5.0	3.65	8.0	5.4	28	0.18	1.083

**Figure 4:**
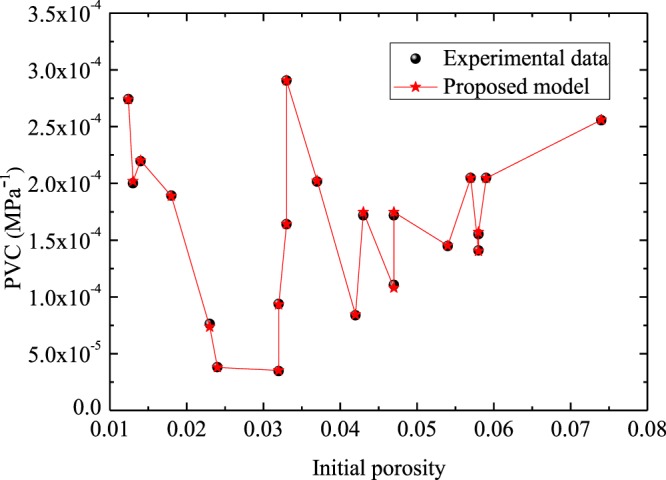
Experimental data^19^ versus the predicted results.

Figure 4 presents the comparison between the predictions of PVC and experimental data reported in the work of Guo *et al*.^19^. In the experiments^19^, PVC tests were conducted on 21 coal samples with an initial maximum pore radius of 0.1 μm. In the calculation, the *r*_*max*0_ was assigned as 0.1 μm, the *r*_*min*0_ was assigned with 0.001 μm, the *E* was assigned with 52 GPa, and the *ν* was assigned as 0.25. Results (Fig. 4) suggest that the predictions are consistent with the measured experimental data^19^. Additionally, simulated results also indicate that the relationship between initial porosity and PVC is not monotonic, suggesting that PVC is correlated not only to ${\phi }_{0}$, but also to other pore structure parameters.

### Parameter influence

**Figure 5:**
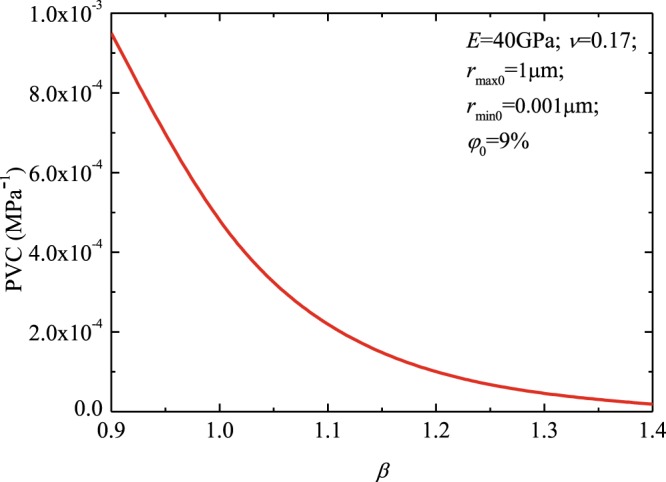
The PVC versus power law index *β*.

Figure 5 presents PVC versus the power law index *β*. In the calculation, the *r*_*max*0_ was 1 μm, the *r*_*min*0_ was 0.001 μm, the initial porosity ${\phi }_{0}$ assigned was 9%, the rock Poisson’s ratio *ν* assigned was 0.17, and the rock elastic modulus *E* assigned was 40 GPa. Figure 5 shows that PVC monotonically decreases as power law index *β* increases^46^.

**Figure 6:**
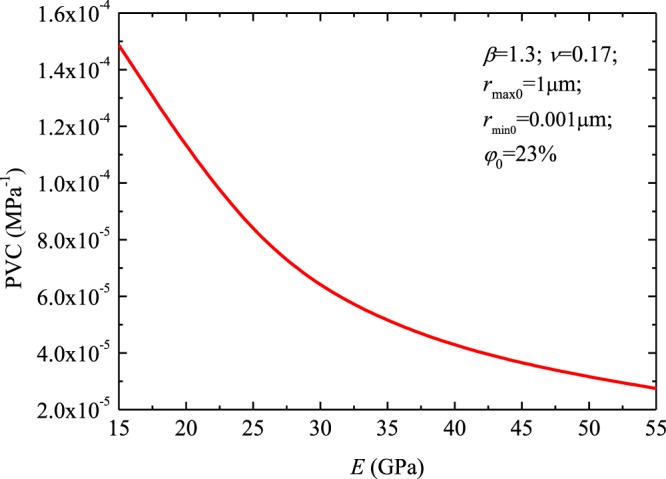
The PVC versus rock elastic modulus.

Figure 6 shows the PVC variation with different rock elastic modulus. For the calculations necessary to assemble this plot, the *r*_*max*0_ was 1 μm, the *r*_*min*0_ was 0.001 μm, the initial porosity ${\phi }_{0}$ assigned was 23%, parameter *ν* was 0.17 and *β* was 1.3. Figure 6 suggests that the PVC decreases as the parameter *E* increases. The main reason for this is that the larger *E* corresponds to the smaller contact surface radius, which leads to the smaller PVC. As a result, reservoir rocks with relative “softer” lithology can yield higher compressibility^13^.

**Figure 7:**
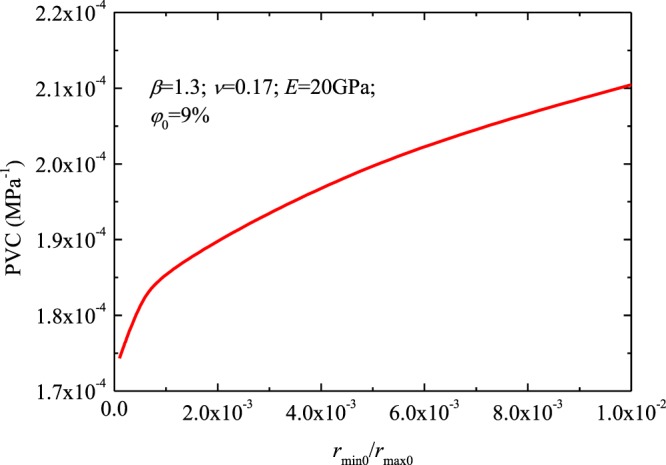
The PVC versus the parameter *r*_*min*0_/*r*_*max*0_.

Figure 7 demonstrates the PVC versus the parameter *r*_*min*0_/*r*_*max*0_. In the calculation, the initial porosity ${\phi }_{0}$ assigned was 9%, parameter *E* assigned was 20 GPa, *ν* assigned was 0.17, and *β* assigned was 1.3. PVC increases with *r*_*min*0_/*r*_*max*0_ (Fig. 7), indicating that smaller pores exhibit lower stress sensitivity than larger pores. Increases of parameter *r*_*min*0_/*r*_*max*0_ (increases in the initial minimum radius *r*_*min*0_) lead to increases of PVC.

## Discussions

### Effect of pore aspect ratio on PVC

It’s well known that the real cross-sectional shapes of pores are complex and highly irregular^9^. Zimmerman^9^ suggested that the influence of pore aspect ratio (i.e., the ratio of pore’s height to its width) on PVC of porous media was significant, which could not be neglected. As stated in the literature^9^, the PVC of an elliptical tubular pore *c*_*pe*_ is

16 $${c}_{pe}={c}_{pr}(\frac{\alpha }{2}+\frac{1}{2\alpha })$$

where *c*_*pr*_ represents the PVC of the circular tubular pore, whose area is the same as that of the elliptical tubular pore (MPa^−1^), and *α* is the pore aspect ratio. Equation 16 shows that, when $\alpha =1$, *c*_*pe*_ is equal to *c*_*pr*_, which is expected. Moreover, *c*_*pe*_ is impossible less than *c*_*pr*_, which illustrates that, when other properties are the same, an elliptical tubular pore is easier to be compressed than a circular tubular pore.

Based on Eqs 9 and 16, the equivalent pore radius of elliptical tubular pores can be modified as

17 $$\{\begin{array}{rcl}{r}_{{maxe}} & = & {r}_{{\max }0}-({r}_{{\max }0}-{r}_{{\max }})\sqrt{(\frac{{\alpha }_{{\max }}}{2}+\frac{1}{2{\alpha }_{{\max }}})}\\ {r}_{{mine}} & = & {r}_{{\min }0}-({r}_{{\min }0}-{r}_{{\min }})\sqrt{(\frac{{\alpha }_{{\min }}}{2}+\frac{1}{2{\alpha }_{{\min }}})}\end{array}$$

where *r*_*maxe*_ represents the equivalent maximum pore radius (μm), *r*_*mine*_ represents the equivalent minimum pore radius (μm), *α*_*max*_ is the pore aspect ratio of the maximum pore, and *α*_*min*_ is the pore aspect ratio of the minimum pore. For the sake of simplicity, the values of pore aspect ratio of each elliptical pore are assumed to be equal. Then, Eq. 17 can be rewritten as

18 $$\{\begin{array}{rcl}{r}_{{maxe}} & = & {r}_{{\max }0}-({r}_{{\max }0}-{r}_{{\max }})\sqrt{(\frac{{\alpha }_{av}}{2}+\frac{1}{2{\alpha }_{av}})}\\ {r}_{{mine}} & = & {r}_{{\min }0}-({r}_{{\min }0}-{r}_{{\min }})\sqrt{(\frac{{\alpha }_{av}}{2}+\frac{1}{2{\alpha }_{av}})}\end{array}$$

where *α*_*av*_ is the average pore aspect ratio of the elliptical pores in the porous media.

Substituting Eq. 18 into Eqs 7 and 10 is rewritten as

19 $$\phi ={({r}_{{\min }0}/{r}_{{\max }0})}^{\tfrac{(2-{D}_{f0}){r}_{{\max }0}}{(3-{D}_{f0}){r}_{{maxe}}-(2-{D}_{f0}){r}_{{\max }0}}}$$

**Figure 8:**
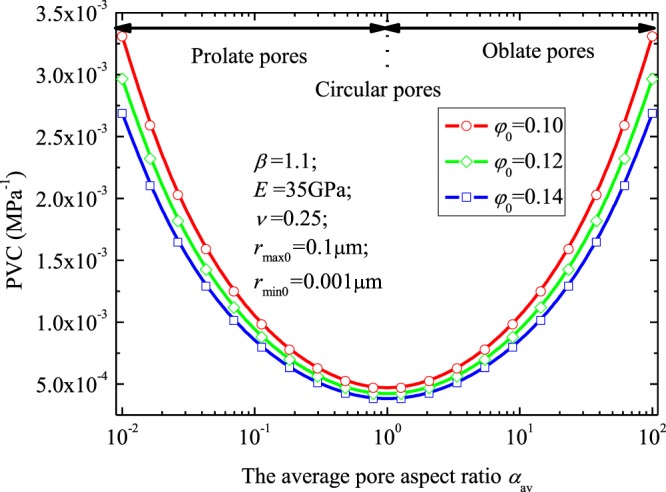
The PVC versus the parameter *α*_*av*_.

Then, with the same method stated above, PVC of porous media accounts for pore aspect ratio can be determined. Figure 8 demonstrates the PVC versus the parameter *α*_*av*_. Results (Fig. 8) suggest that porous media with more elliptical pores (e.g., prolate pores and oblate pores) has a larger value of PVC. Similar results were also shown in the literature^9^.

### Advantages and limitations of the derived model

The analytical model lays a theoretical foundation for predicting the PVC of porous media. With this new analytical solution,the uncertainty in flow through porous media could be reduced and higher data accuracy could be obtained. Moreover, it can be applied to estimate more accurate microstructure parameters of porous media using inverse modeling. However, our derived model is limited to matrix core samples, as it ignores the deformation of joints and fractures. And we are trying to study the PVC of fractured porous media. In addition, the pore aspect ratio distribution of the real pores in porous media is neglected, which may result in the calculation errors.

## Conclusions

A novel analytical model to predict porous media PVC is derived. Predictions deduced from the derived analytical solution agree well with the test results. And the predicted PVC values are robust. Followings are the main conclusions:

1. Porous media PVC is affected by its porous media microstructural parameters and rock lithology. When all the other parameters are fixed, there is a definitive negative correlation between PVC and initial porosity. However, if all the other parameters are inconstant, the relationship between initial porosity and PVC is not monotonic. The blind application of pore compressibility-initial porosity relationships may lead to excessive uncertainty in any analyses based on those relationships. Thus, the specific pore structure parameters for various types of rocks need to be defined through further study to improve the accuracy of the PVC prediction, and in turn production prediction.
2. Porous media with relative “softer” lithology may yield larger PVC. In addition, a smaller value of PVC corresponds to rougher pore surfaces. As rock lithology (e.g., Poisson’s ratio, and elastic modulus) is affected by the rock physical properties (e.g., mineral component, organic matter distribution, and clay content), when making a prediction about PVC, the rock physical properties should be considered.
3. The new analytical model, considering porous media microstructure and rock lithology, is useful for PVC prediction of porous media. It can help to reduce the uncertainty in flow through porous media. What’s more, besides providing theoretical foundations for quantifying PVC of porous media, this new analytical model could also be applied to estimate pore structure parameters of porous media using inverse modeling.

## Data Availability

The datasets generated during and/or analyzed during the current study are available from the corresponding author on reasonable request.
